# Bions: A Family of Biomimetic Mineralo-Organic Complexes Derived from Biological Fluids

**DOI:** 10.1371/journal.pone.0075501

**Published:** 2013-09-25

**Authors:** Cheng-Yeu Wu, Lena Young, David Young, Jan Martel, John D. Young

**Affiliations:** 1 Laboratory of Nanomaterials, Chang Gung University, Gueishan, Taoyuan, Taiwan, Republic of China; 2 Center for Molecular and Clinical Immunology, Chang Gung University, Gueishan, Taoyuan, Taiwan, Republic of China; 3 Research Center of Bacterial Pathogenesis, Chang Gung University, Gueishan, Taoyuan, Taiwan, Republic of China; 4 Department of Organismic and Evolutionary Biology, Harvard University, Cambridge, Massachusetts, United States of America; 5 Department of Materials Science and Engineering, Massachusetts Institute of Technology, Cambridge, Massachusetts, United States of America; 6 Laboratory of Cellular Physiology and Immunology, Rockefeller University, New York, New York, United States of America; 7 Biochemical Engineering Research Center, Ming Chi University of Technology, Taishan, Taipei, Taiwan, Republic of China; University of California, Merced, United States of America

## Abstract

Mineralo-organic nanoparticles form spontaneously in human body fluids when the concentrations of calcium and phosphate ions exceed saturation. We have shown previously that these mineralo-organic nanoparticles possess biomimetic properties and can reproduce the whole phenomenology of the so-called nanobacteria—mineralized entities initially described as the smallest microorganisms on earth. Here, we examine the possibility that various charged elements and ions may form mineral nanoparticles with similar properties in biological fluids. Remarkably, all the elements tested, including sodium, magnesium, aluminum, calcium, manganese, iron, cobalt, nickel, copper, zinc, strontium, and barium form mineralo-organic particles with bacteria-like morphologies and other complex shapes following precipitation with phosphate in body fluids. Upon formation, these mineralo-organic particles, which we term bions, invariably accumulate carbonate apatite during incubation in biological fluids; yet, the particles also incorporate additional elements and thus reflect the ionic milieu in which they form. Bions initially harbor an amorphous mineral phase that gradually converts to crystals in culture. Our results show that serum produces a dual inhibition-seeding effect on bion formation. Using a comprehensive proteomic analysis, we identify a wide range of proteins that bind to these mineral particles during incubation in medium containing serum. The two main binding proteins identified, albumin and fetuin-A, act as both inhibitors and seeders of bions in culture. Notably, bions possess several biomimetic properties, including the possibility to increase in size and number and to be sub-cultured in fresh culture medium. Based on these results, we propose that bions represent biological, mineralo-organic particles that may form in the body under both physiological and pathological homeostasis conditions. These mineralo-organic particles may be part of a physiological cycle that regulates the function, transport and disposal of elements and minerals in the human body.

## Introduction

Mineralo-organic complexes in the form of amorphous and crystalline nanoparticles (NPs) have been observed in various body fluids [1]–[10]. The prevalent forms of minerals that aggregate with organics (proteins, peptides, amino acids, lipids, carbohydrates, and so forth) have been shown to contain predominantly carbonate hydroxyapatite (HAP) structures, which show a tendency to aggregate and to undergo phase transformation. In the process, they assume marked morphological *pleomorphism*, with the spherical NPs flattening out to become spindles, platelets, and films [1]–[10]. These granular shapes are present throughout nature and could represent a more general, and perhaps ubiquitous, cycle of calcium storage, retrieval, deposition, and disposal (see ref. [11] for Ryall’s insightful review on calcium granules). These granules are found in organisms spanning a wide range of phylogenetic complexities [12]–[14].

Our interest in NPs was triggered by a vast amount of literature surrounding the so-called primitive life-forms known as nanobacteria (NB) that have been described as the smallest living symbionts on earth [15]–[22], with claimed implications for a number of disease processes that include Alzheimer’s disease [23], atherosclerosis [24], calciphylaxis [25], cancer [26]–[28], co-infection with HIV [29], ectopic calcification [22], [24], [30]–[37], male infertility [38], periodontal disease [39], polycystic kidney disease [40], prostatitis [41], [42], arthritis [43], [44], and renal stone formation [17]–[21], [45], [46]. NB have also been implicated as potential worldwide airborne pathogens [47], as potential contaminants in vaccines [48], and as infectious agents of disease transmissible through intravenous injection and other forms of inoculation [34]. While controversial, NB have attracted worldwide attention, including the calling for a special workshop by the National Academy of Sciences to address the minimal parameters (and size) for life as permitted under currently known microbiological conditions [49]. The existence of NB, as claimed, has been refuted not only on teleological grounds [50] but also by experimental evidence [1]–[6], [51]–[54]. From these studies, it is now clear that NB are no more than mineralo-organic or mineralo-protein complexes that are formed when precipitating ions like calcium and phosphate come in contact with charged organic moieties. In fact, NB-like forms can be replicated in vitro by using purified proteins and ions [4].

While our studies have disproved the NB hypothesis, it is nonetheless clear that the NPs formed by minerals and organic compounds are no less real, and they have been documented in all the body fluids that we have studied to date, including serum [2], [3], [6], [10] and a number of other body fluids (saliva, urine, cerebrospinal fluid, ascites fluid, synovial joint fluid, pleural effusion) [2], [7]. In addition to the critical role of calcium and phosphate ions in the process of biomineralization, we have noticed that other ions, like magnesium and carbonate, play an important modulating role that affects the speed of formation as well as the final size of the NPs obtained [1], [2].

These preliminary results have prompted us to examine the interaction between a family of other transition group elements and body fluid milieu, all in the context of NP formation as modeled and standardized through our earlier studies [1]–[10]. In particular, we evaluated the outcome of the interaction between charged elements and body fluids, which is relevant to the broader theme of nanotoxicology [55]–[58]. This issue is also relevant for understanding how synthetic NPs interact with body fluids [59]–[67]. In fact, synthetic NPs of various chemical compositions acquire an outer layer of proteins that have been termed “protein corona” [61], [63], [66], [67], and this layer influences the interactions of NPs with cells and other body systems as well as their distribution, retrieval, and disposal in the body [60], [61], [63], [64]. Our studies on carbonate-apatite NPs, on the other hand, describe mineralo-protein complexes formed spontaneously from ingredients (ions and organic compounds) found naturally throughout the body and nature [2], . These apatite particles not only assemble spontaneously under appropriate physiological conditions and are internalized by cells [8], but also interact with immune cells and activate pro-inflammatory responses in a size-dependent manner [10].

It remains to be seen however whether similar mineralo-organic complexes or NPs can be obtained by the simple binding between ions and organics present in biological fluids. Likewise, it remains to be seen whether an entire spectrum of amorphous-to-crystalline phase transformations could be observed for these other ions and minerals similar to what we have observed with carbonate-apatite NPs.

In this context, the present study represents the initial characterization of an entire family of mineralo-organic complexes/NPs that we have previously called *bions* [5]. The data shown here indicate that bions include not only carbonate HAP structures but an entire array of complexes formed from the interaction of ions and organic compounds and that biomimetically resemble complex structures that at times resemble lifeforms.

## Results and Discussion

### General Strategy and Study Outline

Carbonate-apatite NPs form spontaneously in biological fluids through a slow process of nucleation and growth [2]. The study of these mineral particles has been hampered however by the long incubation time required for particle formation and the low amount of particles obtained. In order to circumvent these issues, we have previously developed a simple precipitation method to produce carbonate-apatite NPs rapidly and in sufficient amounts for in-depth analysis [2], [3]. This method recently allowed us to study the physicochemical and biological properties of carbonate-apatite NPs formed in serum in order to understand their formation in body fluids as well as their effects on cultured cells in general [8] and on immune cells in particular [10]. In the present study, we used the precipitation method to examine the fate of various charged elements and ions coming in contact with animal body fluids. We selected a wide range of elements found in the human body and/or in the environment. The elements selected include sodium, magnesium, aluminum, calcium, manganese, iron, cobalt, nickel, copper, zinc, strontium, and barium. They were each added separately to a cell culture medium (i.e., Dulbecco’s modified Eagle’s medium or DMEM) containing a body fluid like serum, followed by the addition of phosphate as a counter ion to induce mineral precipitation. We then examined the morphologies, composition, and properties of the mineral precipitates.

We expected that the precipitation of various elements and ions in biological fluids may result in the formation of biomimetic, mineralo-organic NPs similar to the so-called NB as well as various mineral aggregates or inclusions described earlier in the human body (the term “biomimetic” was used here in the sense that the particles may resemble living microorganisms in terms of morphologies and properties as described in the sections below). We hypothesized further that mineralo-organic NPs (bions) may form normally in the body and play a role in the processing, retrieval and storage of excess elements and ions under both normal physiological and pathological conditions. The formation and fate of bions in the body may thus help to define a novel biological cycle previously overlooked.

### Formation of a Family of Mineralo-Organic NPs in Biological Fluids Displaying Polymorphic Morphologies Resembling Bacteria and Complex Biological Structures

**Figure 1:**
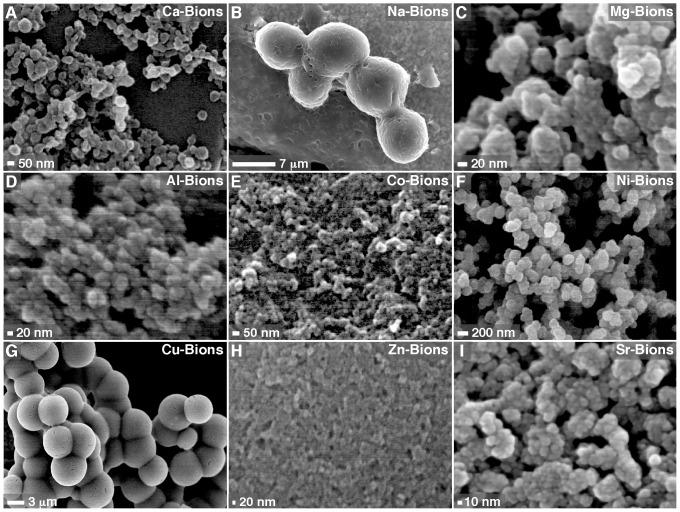
Formation of mineral particles with biomimetic morphologies in biological fluids. The mineral particles shown here, which we refer to as bions, were prepared by adding 1_2_ (A), NaCl (B), MgCl_2_ (C), AlCl_3_ (D), CoCl_2_ (E), NiCl_2_ (F), CuCl_2_ (G), ZnCl_2_ (H) or SrCl_2_ (I) in DMEM containing 10% FBS, followed by addition of 1 mM Na_2_HPO_4_ to induce mineral precipitation. After incubation overnight in cell culture conditions, bions were collected by centrifugation, washed with HEPES buffer and prepared for SEM as described in *Materials and Methods*. Bions formed round, bacteria-like structures at various degrees of aggregation and conversion to crystalline films.

We first prepared carbonate-apatite NPs using the precipitation method described above. Mineral NPs were prepared by adding 1 mM calcium in DMEM containing 10% FBS, followed by addition of 1 mM phosphate to induce mineral formation. The mineralo-organic NPs formed this way—referred also as calcium-based bions or Ca-bions—consisted of particles with a round, bacteria-like shape of relatively homogenous sizes ranging between 20 and 500 nm (Fig. 1A). Ca-bions tend to aggregate and coalesce with time to form biofilm-like formations in culture (Fig. 1A). The entire spectrum of polymorphic morphologies, ranging from small spherical, amorphous NPs to spindles and films, resemble not only NB-like structures described earlier [2]–[4], [6] but also mineral NPs and aggregates detected in mineralized tissues of vertebrates [68]–[70].

Notably, when calcium was replaced with another cation—either sodium, magnesium, aluminum, cobalt, nickel, copper, zinc, or strontium—prior to the addition of phosphate, the mineral precipitates also displayed round mineral particles with bacteria-like morphologies (Fig. 1B-I). The bions formed showed relatively homogenous sizes within any given mineral sample but they varied in size depending on the cations used. For instance, cations like magnesium, aluminum, cobalt, nickel, zinc and strontium mostly produced small NPs (10–300 nm), whereas sodium and copper produced large particles with diameters of several micrometers (1–10 µm, Fig. 1B-I). Moreover, bions formed by cations like sodium (Fig. 1B) gave a crystalline appearance while bions formed from copper (Fig. 1G) clearly displayed a metallic tint, in contrast to other bions like Ca-bions (Fig. 1A) that produced mainly amorphous spheres and aggregates. Bions also showed a tendency to aggregate and form colonies after incubation in cell culture conditions.

**Figure 2:**
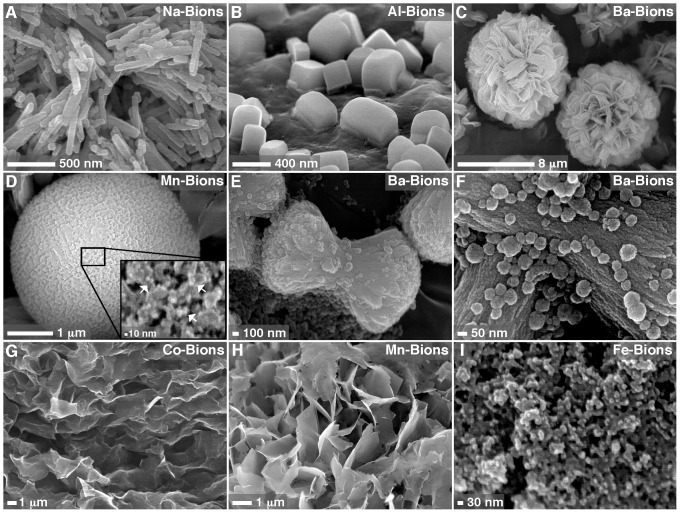
Bions form various biomimetic morphologies and complex structures in biological fluids. Bions were prepared by adding 1(A), AlCl_3_ (B)_,_ BaCl_2_ (C, E, and F), or MnCl_2_ (D) in DMEM containing 10% FBS (A, C, and D) or 10% HS (B, E, and F), followed by addition of 1 mM Na_2_HPO_4_ to induce mineral precipitation. In (A), 1 mM Na_2_CO_3_ was also added before addition of Na_2_HPO_4_. Bions were incubated in cell culture conditions overnight, collected by centrifugation, washed with HEPES buffer, and prepared for SEM. Bions formed various morphologies, including shapes similar to bacillus bacteria (A), mushrooms (B), and flowers (C), as well as round, cell-like formations apparently formed of smaller, round NPs (D; inset, NPs denoted by white arrows). Complex structures similar to dumbbells and muscle fibers (E and F) were also observed in some bion samples. (G) Addition of 1 mM CoCl_2_ and Na_2_HPO_4_ each in water containing 10% FBS produced mineral NPs that quickly converted into crystalline biofilm-like structures. (H) When MnCl_2_ and Na_2_HPO_4_ were added at 1 mM each in DMEM alone without a body fluid, a mineral precipitate showing a flaky appearance and sharp edges was observed. (I) Addition of 1 mM FeCl_2_ and Na_2_HPO_4_ each in water formed round Fe-bions of small sizes. The samples shown in (G–I) were incubated overnight before preparation for SEM.

In addition to forming round NPs, bions also formed a variety of striking morphologies, including shapes similar to bacillus bacteria, mushrooms, and flowers (Fig. 2A, B, and C, respectively). We also observed the formation of large spheres that appeared to consist of smaller NP subunits (Fig. 2D, inset). These structures are reminiscent of the so-called mesocrystals—imperfect-or-composite crystalline materials consisting of multiple oriented nanocrystals embedded in an amorphous matrix [71], [72]. Other complex three-dimensional topologies were also observed, including structures resembling dumbbells and muscle fibers (Fig. 2E and F, respectively). These polymorphic morphologies were quite reproducible in our hands and they appeared to represent morphological signatures for each of the cations present in the bion complex (Fig. 2).

When bions were prepared with serum dissolved into water instead of DMEM, we noticed that some cations like cobalt produced round mineral NPs (Co-bions) that rapidly converted to crystalline morphologies with sharp edges and needle-like structures following overnight incubation (Fig. 2G; compare this morphology with that seen with Co-bions formed in DMEM as shown in Fig. 1E). This particle-to-film conversion had been previously described for calcium-based mineralo-organic NPs, and the conversion had been shown to be delayed by organic moieties present in body fluids and cell culture media [2], [10]. The observations presented here suggested that this particle-to-film conversion also occurs for bions.

The role of serum and incubation time during bion formation was also assessed. In the absence of serum and under the conditions used in our study, cations like manganese produced mineral aggregates and biofilm-like structures with crystalline morphologies (Fig. 2H). This coalescence of mineral aggregates followed by crystalline transformation into films or mattresses was enhanced by high concentrations of cations and phosphate as well as by prolonged incubation in cell culture conditions (data not shown), similar to what we had observed earlier for Ca-bions [2]–[10]. Earlier, we had shown that serum could modulate the nucleation and phase transformation of Ca-bions under the conditions studied, with low amounts of serum (≤3% for human serum, or HS, and ≤1% for FBS) favoring mineral aggregation and amorphous-to-crystalline phase transformation while higher amounts of serum favored instead NP nucleation with amorphous disposition in the shape of spheres [2], [10]. This nucleation of NPs and phase-inhibition had earlier been shown to be mimicked by proteins like albumin and fetuin-A [4] but it could be retained using γ-irradiated serum [6] suggesting the participation of other organic constituents including smaller organic moieties. While organic moieties clearly favor the formation of round bions, one notable exception is iron which produced granular particles even in the absence of serum (Fig. 2I). This observation is consistent with previous results [1], [2], [9], [73], [74] which showed that some precipitating ions like magnesium and carbonate may modulate particle formation by favoring the formation of small spherical NPs similar to the Fe-bions described here.

**Figure 3:**
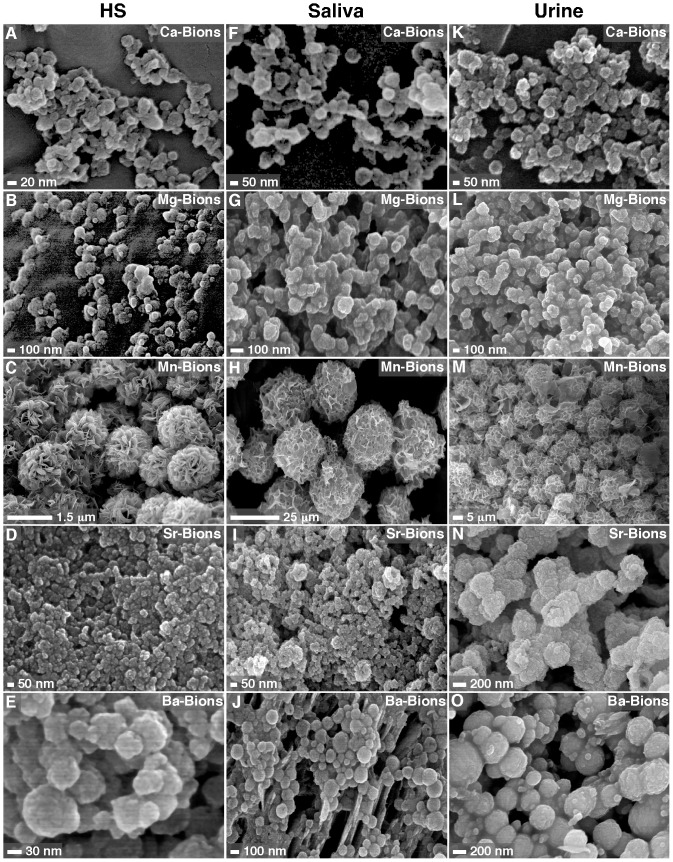
Bions form in various human body fluids. Bions were prepared by adding 1_2_, MgCl_2_, MnCl_2_, SrCl_2_ or BaCl_2_ in DMEM containing 10% HS (A-E), saliva (F-J), or urine (K-O), followed by addition of 1 mM Na_2_HPO_4_. After incubation overnight in cell culture conditions, bions were collected by centrifugation, washed with HEPES buffer, and prepared for SEM. Bions produced in various body fluids displayed bacteria-like morphologies at different stages of aggregation and conversion to biofilm-like structures.

In previous studies [2], [7], we showed that body fluids besides serum, including saliva, urine, cerebrospinal fluid, ascites, pleural effusion, and synovial fluid, could support the formation of Ca-bions. These body fluids were studied in the presence of other cations in order to assess the formation of bions. The results for saliva and urine collected from healthy human individuals are shown and compared with those obtained with HS (Fig. 3). Briefly, body fluids obtained from healthy subjects were inoculated with the specified cations prior to the addition of phosphate and the mineral precipitates were collected and examined by SEM. In the presence of 10% HS in DMEM, the cations tested formed round bions displaying different degrees of coalescence and with striking individual morphologies when mixed with phosphate at 1 mM each (Fig. 3A-E). Both saliva (Fig. 3F-J) and urine (Fig. 3K-O) produced similar spherical bions in various sizes and degrees of crystallization and aggregation when challenged with cations and phosphate under the same conditions. The cation used seemed to modulate the morphology of the bions seen, as exemplified by the large spheres with distinct surface folds and semi-crystalline appearance obtained with Mn (Fig. 3C, H, and M). Other human body fluids (cerebrospinal fluid, ascites, pleural effusion, broncho-alveolar lavage, and synovial fluid) also yielded bions that varied in morphology depending on the amounts of ions and body fluids used as well as the length of incubation, with higher amounts of ions, lower amounts of fluids, and longer incubations favoring crystallization and aggregation (data not shown). The morphologies of the resultant bions did not seem to change noticeably with the particular species of the body fluid used, as exemplified by the data obtained for HS (Fig. 3A-E) versus FBS (Fig. 1). To date, all body fluids tested, be they of human or animal origin, have been shown to interact with cations and phosphate to form distinct looking bions that then undergo amorphous-to-crystalline transformation with time. In this sense, the formation of biomimetic bions appears to be a general phenomenon in nature resulting from the interaction of precipitating ions with an organic milieu.

**Figure 4:**
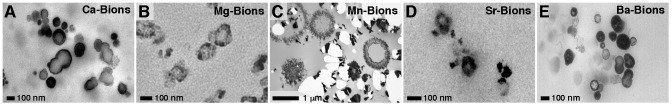
Thin-section TEM observations of bions derived from biological fluids. Bions were prepared by adding 1_2_ (A), MgCl_2_ (B), MnCl_2_ (C), SrCl_2_ (D) or BaCl_2_ (E) in DMEM containing 10% FBS, prior to addition of 1 mM Na_2_HPO_4_. The particles were incubated overnight in cell culture conditions, collected by centrifugation, and washed with HEPES buffer. Thin-sections were observed under TEM without staining. Bions consisted of round bacteria-like formations with a wall and a central cavity. Solid, electron-dense, mineral particles were also noted.

To examine the internal structure of bions, we prepared thin sections of the mineral samples formed in DMEM-FBS and observed them by transmission electron microscopy (TEM) without fixation or staining. Thin sections of Ca-bions revealed small, round particles with electron-dense walls and hollow cavities as well as solid, electron-dense spheres (Fig. 4A). These morphologies resemble the images obtained earlier [3], [4] that also revealed the propensity for Ca-bions to form multi-lamellar spheres with increased concentration of fluids or proteins. Thin sections of bions derived from the other cations also showed a mixture of mineral particles with either electron-dense walls or a solid mineral structure (Fig. 4B-E). In addition to these morphologies, Mn-bions also produced large, electron-dense rings with crystalline surfaces and diameters of several micrometers (Fig. 4C; notice that the morphology and size of these particles were consistent with the SEM observations made for these same Mn-bions in DMEM-HS and shown in Fig. 3C).

Together, these observations demonstrate that a broad variety of charged ions and compounds can form small, round, mineral structures when they precipitate in biological fluids. The mineral particles produced this way become pleomorphic and tend to aggregate and crystallize to form biofilms that may biomimetically resemble lifeforms including complex tissue-like structures—characteristics that are similar to the previous observations made for the mineral NPs observed in human tissues [68]–[70]. Furthermore, the body fluids appear to provide the required organic moieties needed to form and stabilize bions.

### Chemical Composition of Bions Derived from Biological Fluids in the Presence of Various Precipitating Cations

**Figure 5:**
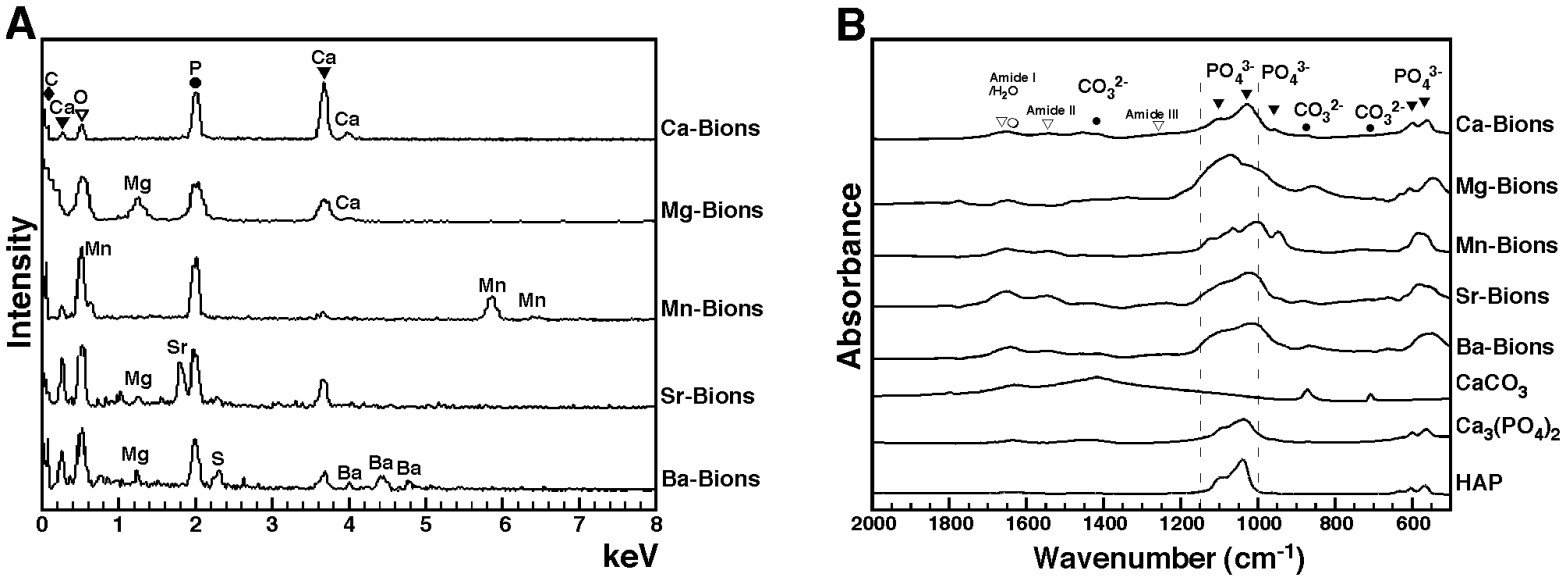
Elemental and chemical compositions of bions formed in biological fluids. (A) EDX spectra of bions prepared as in Fig. 4 showed peaks of carbon, calcium, oxygen, and phosphorus, consistent with the presence of carbonate apatite. Peaks corresponding to the charged cations added were also noted (i.e., Mg^2+^, Mn^2+^, Sr^2+^, or Ba^2+^). The peaks present in every sample were labeled with black geometrical figures on top for clarity. The oxygen peak, which superimposed with the Mn peak in the spectrum of Mn-bions, was also found in all samples, and was labeled with an inverted white triangle. The following calcium:phosphorus atomic ratios were observed: Ca-bions, 1.54; Mg-bions, 0.76; Mn-bions, 0.11; Sr-bions, 0.61; Ba-bions, 0.37. The ratios of added cations to phosphorus were as follows: Mg:P, 0.63; Mn:P, 1.05; Sr:P, 0.41; Ba:P, 0.51. (B) FTIR analysis of bions prepared as in Fig. 4 revealed peaks of phosphate (575 cm^−1^, 605 cm^−1^, 960 cm^−1^, and 1,000−1,150 cm^−1^) and carbonate (875 cm^−1^ and 1,400−1,430 cm^−1^). Low peaks corresponding to amide I/H_2_O, amide II, and amide III at 1,660 cm^−1^, 1,550 cm^−1^, and 1,250 cm^−1^, respectively, were also observed in some samples due to the presence of water and serum proteins. Representative spectra of CaCO_3_, Ca_3_(PO_4_)_2_ and HAP controls were included for comparison.

Previous studies had shown that the mineralo-organic NPs which spontaneously form in body fluids consist of carbonate HAP [2], [3], [8], a mineral similar to the one found in bones and calcified deposits in humans [75], [76]. We first determined the elemental composition of the bions studied here using energy-dispersive X-ray spectroscopy (EDX). This analysis showed that bions obtained following addition of any of the cations and phosphate showed peaks of carbon (C), calcium (Ca), oxygen (O), and phosphorus (P; Fig. 5A). It should be pointed out that calcium is normally present in DMEM at a concentration of 1.8 mM, a fact which should explain its presence in all our bion samples despite the fact that no exogenous calcium was added to our bion preparations except in the case of Ca-bions (Fig. 5A). In addition to showing peaks indicative of carbonate HAP, the EDX spectra of bions revealed the presence of the main precipitating cations used (Mg^2+^, Mn^2+^, Sr^2+^, or Ba^2+^), suggesting that these became associated with the carbonate HAP matrix found in the precipitates (Fig. 5A). Other cations normally present in DMEM (see *Materials and Methods* for a complete list) also appear to become incorporated into the various bion samples, albeit at much lower levels and without experimental consistency (Fig. 5A; see for instance the presence of magnesium in Mg-bions, Sr-bions and Ba-bions and the presence of sulfur in Ba-bions). Accordingly, previous studies had shown that the calcium ions present in both calcium phosphate and carbonate-HAP minerals can be substituted with various cations like magnesium and zinc [77]. Our own earlier experiments had revealed the modulation of carbonate HAP nucleation by magnesium [2]. These observations illustrate the constant HAP composition of bions and the possibility for these particles to incorporate other elements present in solution.

As evidenced from the EDX data, we observed that the calcium:phosphorus (Ca:P) ratio of bion specimens ranged from 0.11 to 1.54 (Fig. 5A), an observation consistent with the variable ratios observed earlier with in vitro HAP preparations seen at various stages of crystallization [2] as well as with bone minerals studied at various stages of apatite formation [78], [79]. For comparison, the ratios of the other cations to phosphorus ranged between 0.41 and 1.05 (Fig. 5A, Mg:P, Mn:P, Sr:P, or Ba:P), all of which are consistent with the likelihood that the cations used in our experiments (i.e., Mg^2+^, Mn^2+^, Sr^2+^, or Ba^2+^) have effectively replaced calcium in the final bion composition subjected to EDX analysis.

We also used Fourier transform infrared spectroscopy (FTIR) to determine the main functional chemical groups associated with bions. FTIR spectra of the various bions studied here revealed peaks of carbonate at 875 cm^−1^ and 1,400−1,430 cm^−1^ and phosphate at 575 cm^−1^, 605 cm^−1^, 960 cm^−1^, and 1,000−1,150 cm^−1^ (Fig. 5B; compare the bion spectra with those of commercially available CaCO_3_, Ca_3_(PO_4_)_2_ and HAP used as reference compounds; see also refs. [80]–[83]). This FTIR analysis largely complemented the EDX results shown above and further confirmed that the bions prepared here contained a mineral phase of carbonate HAP. The FTIR spectra also displayed low peaks corresponding to amide I, II, and III at 1,660 cm^−1^, 1,550 cm^−1^, and 1,250 cm^−1^, respectively (Fig. 5B). These peaks can be attributed to the presence of serum proteins in bions, in line with earlier studies [75], [84].

In the presence of body fluids, cations and carbonate phosphates are seen here to form precipitating HAP complexes that in turn produced biomimetic structures with varying degrees of crystallization and topological complexities. Our results demonstrate that these various cations become incorporated with the resultant precipitating mineral particles. This co-precipitation with HAP suggests that the composition of bions may vary according to the ionic composition of the milieu in which they form. Of note, the carbonate HAP composition of these mineral particles resembles that of the particles formed spontaneously in biological fluids [2], [5] and human tissues [68]–[70]. These findings suggest further that bions may represent a family of closely related mineralo-organic complexes that incorporate various ions and organic moieties and that assume different degrees of crystallization and morphological pleomorphism—a hypothesis that we have put to test next.

### Bions Undergo Amorphous-to-Crystalline Mineral Conversion in Cell Culture Conditions

The formation of bions in the form of biomimetic, mineralo-organic NPs requires an amorphous mineral phase which is seemingly stabilized by calcification inhibitors like competing ions, proteins, and organic molecules present in body fluids [1], [2], [9]. This amorphous mineral phase has been shown to manifest with round particle shape and smooth surface morphologies similar to those of bacteria, and they in turn contrast markedly with the geometrical shapes, sharp edges and faceted surfaces of crystalline minerals [2]. Depending on the incubation time, ion input, and changes in culture conditions, there is a gradual transformation of the unstable amorphous mineral phase to a more thermodynamically favorable crystalline phase which in turn is associated with distinctive and recognizable crystalline characteristics. We have shown earlier that the conversion of amorphous mineral NPs to crystalline phases occurs in a matter of minutes to weeks depending on the culture conditions used [2].

**Figure 6:**
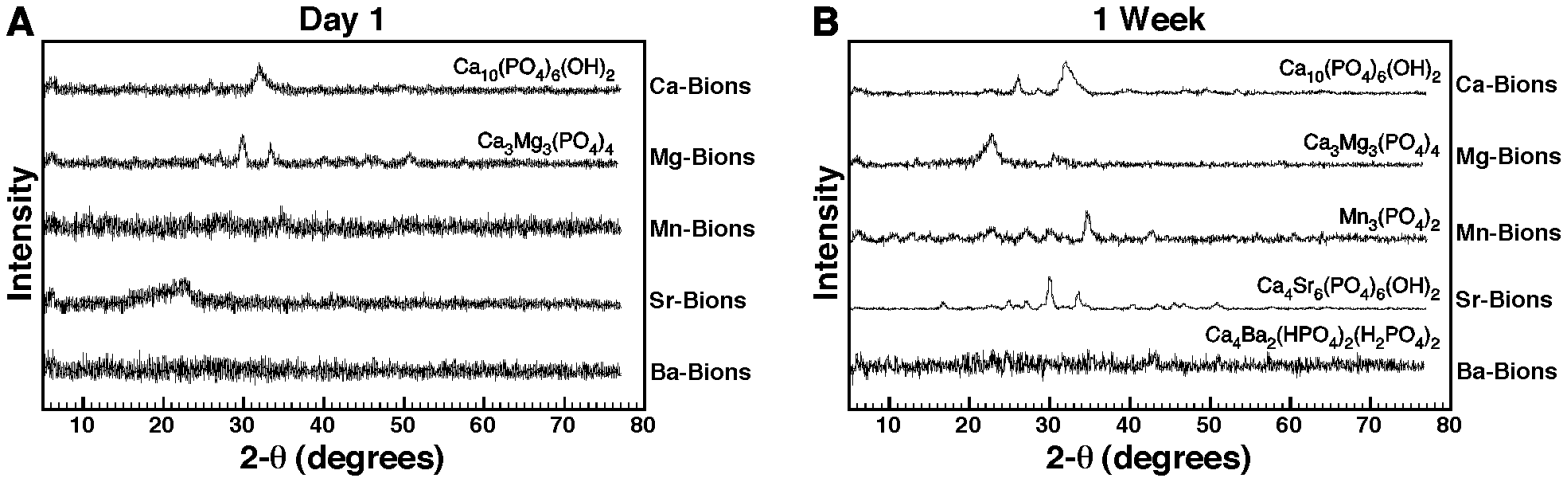
Amorphous-to-crystalline mineral conversion of bions in biological fluids. Bions prepared as in Fig. 4 were incubated for either 1 day (A) or 1 week (B) in cell culture conditions, prior to preparation for XRD analysis. In (A), low, broad peaks corresponding to Ca_10_(PO_4_)_6_(OH)_2_ and Ca_3_Mg_3_(PO_4_)_4_ were noted for Ca-bions and Mg-bions, respectively, whereas the other bion samples produced amorphous spectra. (B) One-week old bions produced sharp peaks corresponding to Ca_10_(PO_4_)_6_(OH)_2_ for Ca-bions, Ca_3_Mg_3_(PO_4_)_4_ for Mg-bions, Mn_3_(PO_4_)_2_ for Mn-bions, Ca_4_Sr_6_(PO_4_)_6_(OH)_2_ for Sr-bions, and Ca_4_Ba_2_(HPO_4_)_2_(H_2_PO_4_)_2_ for Ba-bions. These results suggest that bions initially form an amorphous mineral phase that gradually converts to crystalline mineral during incubation.

Using powder X-ray diffraction analysis (XRD), we first observed that calcium-based bions incubated for one day produced low, crystalline peaks, suggesting the presence of a predominant amorphous mineral phase with a low content of crystals at this early stage (Fig. 6A, Ca-Bions). The low-intensity peaks were attributed by comparing with known chemical compounds in the database to HAP with the chemical formula Ca_10_(PO_4_)_6_(OH)_2_, an observation that would also be consistent with the EDX and FTIR analyses presented above. On the other hand, carbonate was not annotated separately in the XRD analysis performed here since the main signals for HAP and carbonate HAP on the 2-θ scale are indistinguishable as noted previously [2], [3]. Likewise, the bion samples prepared in the presence of magnesium also produced low, broad peaks which in this case matched with those attributable to Ca_3_Mg_3_(PO_4_)_4_ (Fig. 6A, Mg-Bions). On the other hand and under the conditions studied, Mn-bions, Sr-bions and Ba-bions did not produce any consistent or identifiable peaks, suggesting the presence of predominant amorphous mineral phases for these samples collected after 1 day of incubation (Fig. 6A, Mn-Bions, Sr-bions, and Ba-Bions).

The same bion samples were also incubated for one week in cell culture conditions and re-examined by XRD. Bion samples now showed a higher number of distinct and sharp peaks (Fig. 6; for each bion sample, compare the spectrum in B with that obtained after 1 day of incubation as shown in A). These same bion samples after one week of incubation produced diffraction peaks that could be readily identified as the distinct HAP compounds shown in Fig. 6B. Like the EDX and FTIR spectra, the XRD analysis yielded distinct spectra and chemical compositions that matched with the cations used (i.e., Ca^2+^, Mg^2+^, Mn^2+^, Sr^2+^, or Ba^2+^), indicating that they become integral components of the precipitating mineral complexes formed.

Our data also indicate an amorphous-to-crystalline conversion that takes time to proceed and that reaches varying degrees of completion even when the same amounts of cations are present (as evidenced for example by the slower conversion of Ba-bions to crystalline minerals seen in Fig. 6B). In this respect, we noticed that the rate and the extent of this same amorphous-to-crystalline conversion seemed to vary with the size of the cation added to the precipitating solution. Smaller cations like calcium and magnesium produced crystalline materials with faster kinetics (Fig. 6A) whereas larger cations like barium persisted in amorphous state much longer, taking weeks to produce significant crystalline conversion.

We conclude that bions are initially amorphous following precipitation in biological fluids, likely due to the presence and binding of organic compounds, and this hypothesis will be further addressed next. This amorphous mineral phase corresponds to the round morphologies of bions initially formed (Figs. 1, 2, 3, 4; see also refs. [1], [2], [9], [85]). In culture, and presumably in body fluids, amorphous bions may undergo a time-dependent conversion to a crystalline mineral phase, a process similar to what was observed previously for calcium-carbonate-phosphate NPs prepared in body fluids [2].

### Serum Produces a Dual Inhibition-Seeding Effect on Bion Formation

We observed earlier that serum as well as all body fluids studied to date appeared to contain components that favor the formation of Ca-bions when challenged with precipitating ions like calcium and phosphate [2]. Paradoxically, in the case of serum, this body fluid is required in most instances for Ca-bion formation but this seeding activity appeared optimal only at low amounts of serum, with higher amounts appearing to delay Ca-bion formation in the presence of the same amounts of precipitating ions [2]. These observations led us to propose a dual inhibition-seeding model in which serum is deemed to propitiate the nucleation of organo-ionic complexes that are nonetheless aborted in growth and propagation by the same serum factors [4], [5]. According to this model, only when the same organo-ionic complexes are overwhelmed by precipitating ions at the expense of organic moieties do particle growth and crystalline conversion occur. Without this ionic vs. organic imbalance, the same organo-mineralo complexes would tend to stay as small, amorphous NPs with well-defined protein corona characteristic of the body fluid milieu from which bions originate [10]. With prolonged incubation and the continued presence of precipitating ions, it is clear that the amorphous-to-crystalline conversion ensues spontaneously, favoring an increase in size and number. In this sense also, our model calls for every one of the organo-ionic complexes to behave as potential mineral seeders that will eventually grow in size under favorable conditions [2], [4], [5], [10].

**Figure 7:**
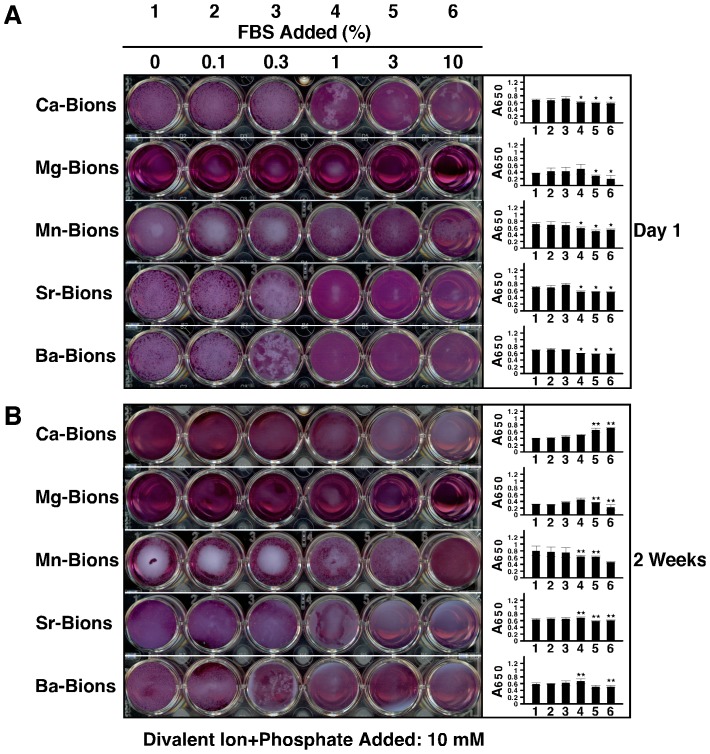
Serum produces a dual inhibition-seeding effect on bion formation. Bions were prepared in 24-well plates by adding 10 mM CaCl_2_, MgCl_2_, MnCl_2_, SrCl_2_ or BaCl_2_ in DMEM containing 0.1–10% FBS, followed by addition of 10 mM Na_2_HPO_4_ to induce mineral precipitation. Each bion preparation consisted of a total of 1 ml. The plates were incubated in cell culture conditions for one day (A) or two weeks (B), prior to photography and spectrophotometry measurements at a wavelength of 650 nm (A_650_). While FBS inhibited bion formation in a dose-dependent manner at day 1, the inhibition was gradually overcome with time as seen by the additional bion precipitation observed after 2 weeks (compare “Day 1” with “2 Weeks”). *P < 0.05, versus well 1 of the same row; **P < 0.05, versus the same well at Day 1.

In the context of the present study, we examined whether the dual inhibition-seeding role of serum could be generalized for other types of precipitating cations insofar as bion formation is concerned. We adopted the simple photographic and absorbance method adapted to 24 well-plates developed earlier [2] in order to study bion formation and precipitation in the presence of various cations and other treatment conditions. In addition to visual inspection and photography, we monitored mineral particle formation by measuring absorbance at 650 nm (Fig. 7, insets, A_650_).

As expected, serum produced a dose-dependent inhibitory effect on Ca-bion formation induced by addition of calcium and phosphate at 10 mM each in DMEM (Fig. 7A). Notably, serum also inhibited in a dose-dependent manner bion formation induced by the addition of 10 mM of manganese, strontium, or barium (Fig. 7A). Inhibition of bion formation by serum was seen at levels comparable to those seen earlier [2] and here for Ca-bions (Fig. 7A). That is, as little as 0.3-1% serum produced significant inhibitory effects against the relatively high amounts of precipitating ions used in our experiments (i.e., 10 mM). Of the cations studied, Mg alone stood as an exception, with very little precipitation seen to begin with, either in the presence or absence of serum (Fig. 7A, Mg-bions). This same effect was noticed earlier [2], and it was attributed to the predominantly inhibitory, rather than seeding, effects of magnesium on HAP formation.

To have a better picture of the dual inhibition-seeding effect of serum on the bion formation induced by cations other than calcium, we incubated the same bion samples for two weeks in cell culture conditions. After this incubation period and in line with what we observed earlier with Ca-bions [2]–[4], we noticed that optically dense bions increased markedly in quantity (Fig. 7B), confirming that the largely inhibitory effect of serum seen on the 1^st^ day of incubation was lost or overwhelmed by 2 weeks, shifting by then to a net seeding effect. This delayed seeding effect of serum was observed not only for Ca-bions but also for the particles prepared from other cations (i.e., Mg^2+^, Mn^2+^, Sr^2+^, or Ba^2+^). Essentially, the dose-dependent inhibitory pattern produced by serum was shifted to the right after 2 weeks (Fig. 7B; compare with Fig. 7A), with serum producing inhibitory effects only at concentrations higher than 3%. At 2-week incubation, even Mg-bions displayed measurable seeding and a rightward-shift in seeding inhibition (Fig. 7B). We conclude from these results that the dual inhibition-seeding effect of serum on bion formation can be generalized to cations other than calcium, suggesting a universal model for bion formation that is predicated to the presence of organic molecules present in various body compartments.

### Identification of Bion-Binding Proteins

We have shown previously that carbonate apatite NPs (Ca-bions) prepared in serum bind to a wide range of proteins that can be identified and categorized by a rapid proteomic methodology that we have developed to study the protein composition of NPs [7]. These proteins include for example calcification inhibitors, coagulation factors, complement proteins, immune regulators, protease inhibitors, and lipid/molecule carriers [7]. Likewise, the nature of the proteins that coat man-made synthetic NPs is believed to influence the effects and the distribution of these same particles in vivo [61], [63], [67]. For the bions studied here, we first sought to determine their protein profile after separation by sodium dodecyl sulfate-polyacrylamide gel electrophoresis (SDS-PAGE). For these experiments, bions were precipitated by adding 10 mM of the various cations and phosphate to DMEM containing 5% FBS, followed by washing and treatment with ethylene-diamine-tetra-acetic acid (EDTA) and SDS to release particle-bound proteins. Aliquots of the released proteins were then separated by gel electrophoresis under denaturing and reducing conditions, followed by staining with Coomassie blue. Mg-bions were prepared on the other hand with 20 mM of Mg instead of 10 mM due to the low amount of precipitation observed for this cation.

**Figure 8:**
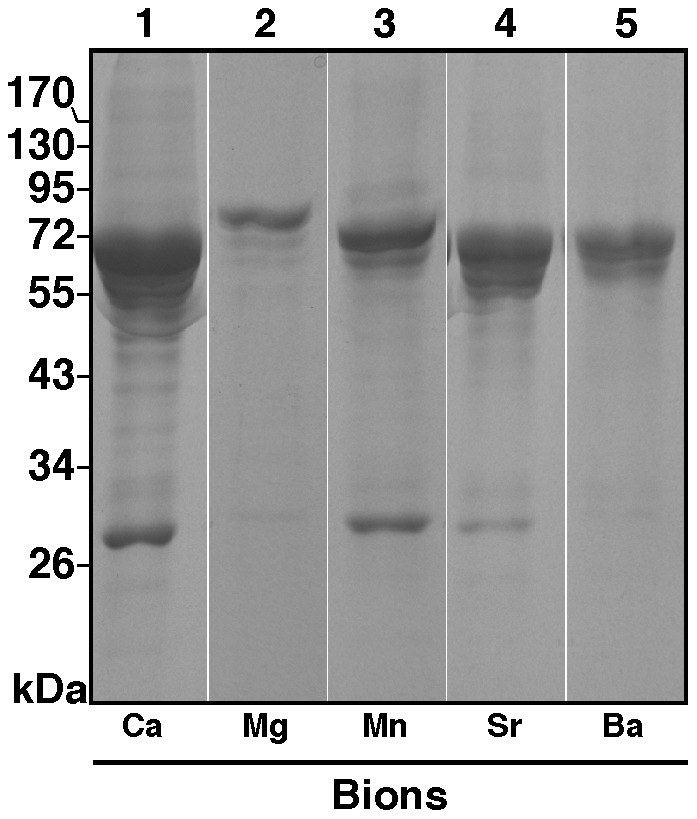
Protein profiles of bions derived from serum. Bions were prepared by adding 10_2_, MnCl_2_, SrCl_2_ or BaCl_2_ in DMEM containing 5% FBS, followed by addition of 10 mM Na_2_HPO_4_ to induce bion formation. A final volume of 1 ml was used. Mg-bions were prepared by adding 20 mM MgCl_2_ due to the lower amount of precipitation observed for this cation. The particles were incubated in cell culture conditions for two days. Following incubation, the particles were washed and treated with 0.1 mM EDTA. The protein samples were prepared for SDS-PAGE under denaturing and reducing conditions (see *Materials and Methods*).

The protein profiles of bions as visualized by Coomassie blue staining typically consisted of a few prominent bands at 45–75 kDa and a more promiment single band at 28–30 kDa (Fig. 8, lanes 1–5). These contrast with a much broader smear of bands seen with Ca-bions (Fig. 8, lane 1). Compared to the other bions, Ba-bions produced weaker protein bands (Fig. 8, lane 5) while Mg-bions contained even fewer visible bands (Fig. 8, lane 2); noticeably, the 28–30 kDa protein band was largely missing from these two bion groups. These results likely indicate differences in protein-affinities that are likely to be specific to the cations used. Overall, however, the protein profiles of bions were surprisingly similar to the ones observed previously for carbonate apatite NPs that were prepared with calcium and phosphate ions under similar conditions [2], [7].

In order to better define the nature of these bion-binding proteins, we used the rapid proteomic method established previously [7]. Our proteomic approach is based on the use of in-solution trypsin digestion, liquid chromatography (LC), and tandem mass spectrometry (MS) analysis. The proteins identified this way were ranked by relative abundance using the number of MS/MS spectra for each protein (Table 1).

**Table 1:** Bion-binding proteins identified by LC-MS/MS and ranked by spectral counting.

				Bions				
No.	Protein identified	Accession Number	MW	Ca^2+^	Mg^2+^	Mn^2+^	Sr^2+^	Ba^2+^
1	Fetuin-A	FETUA_BOVIN	38	373.4	154.8	150.4	495.1	490.3
2	Albumin	ALBU_BOVIN	69	232.5	339.7	322.9	97.2	82.9
3	Apolipoprotein A-I	APOA1_BOVIN	30	16.2	21.5	50.5	7.9	9.1
4	Prothrombin	THRB_BOVIN	71	14.1	17.2	18.2	29.5	61.7
5	Complement C3	CO3_BOVIN	187	26.1	21.5	62.1	12.8	13.1
6	Hemoglobin fetal subunit beta	HBBF_BOVIN	16	10.6	–	17.7	7.9	7.1
7	Alpha-1-antiproteinase	A1AT_BOVIN	46	7.8	17.2	22.7	3.9	6.1
8	Vitamin K-dependent protein S	PROS_BOVIN	75	3.5	21.5	3.5	27.5	35.4
9	Serotransferrin	TRFE_BOVIN	78	6.3	34.4	18.7	–	–
10	Coagulation factor X	FA10_BOVIN	55	10.6	–	3.5	19.6	17.2
11	Coagulation factor IX	FA9_BOVIN	47	4.9	–	4.0	14.7	17.2
12	Apolipoprotein A-II	APOA2_BOVIN	11	4.9	–	12.6	1.0	1.0
13	Vitamin D-binding protein	VTDB_BOVIN	53	3.5	8.6	12.6	1.0	–
14	Vitamin K-dependent protein C	PROC_BOVIN	51	1.4	8.6	1.5	12.8	16.2
15	Hemoglobin subunit alpha	HBA_BOVIN	15	7.0	–	10.1	2.0	2.0
16	Adiponectin	ADIPO_BOVIN	26	3.5	8.6	7.1	9.8	3.0
17	Coagulation factor V	FA5_BOVIN	249	4.2	–	5.6	5.9	3.0
18	Alpha-2-macroglobulin	A2MG_BOVIN	168	1.4	12.9	3.5	–	2.0
19	Antithrombin-III	ANT3_BOVIN	52	2.1	–	9.1	2.9	3.0
20	Alpha-fetoprotein	FETA_BOVIN	69	2.1	–	20.7	–	–
21	Heat shock protein HSP 90-alpha	HS90A_BOVIN	85	6.3	–	1.5	11.8	12.1
22	Complement factor H	CFAH_BOVIN	140	4.2	–	12.6	–	–
23	Thrombospondin-1	TSP1_BOVIN	130	0.7	–	5.6	2.0	1.0
24	Apolipoprotein M	APOM_PIG	21	–	–	2.5	–	–
25	Secreted phosphoprotein 24	SPP24_BOVIN	23	1.4	–	–	2.9	–
26	Apolipoprotein E	APOE_BOVIN	36	–	8.6	2.0	–	–
27	Osteopontin	OSTP_BOVIN	31	1.4	–	1.0	2.0	2.0
28	Alpha-2-antiplasmin	A2AP_BOVIN	55	0.7	4.3	1.0	2.0	1.0
29	Protein disulfide-isomerase	PDIA1_BOVIN	57	1.4	–	–	2.9	–
30	Complement component C9	CO9_BOVIN	62	–	–	2.5	–	–
31	Vitamin K-dependent protein Z	PROZ_BOVIN	43	–	–	–	2.0	1.0
32	Complement C4 (Fragments)	CO4_BOVIN	102	2.8	–	2.5	–	3.0
33	Coagulation factor VII	FA7_BOVIN	49	–	–	–	2.0	2.0
34	Endoplasmin	ENPL_BOVIN (+2)	92	1.4	–	–	–	3.0
35	Apolipoprotein C-III	APOC3_BOVIN	11	1.4	–	1.0	–	–
36	Glucosidase 2 subunit beta	GLU2B_BOVIN	60	–	–	0.5	2.0	–
37	Mannose-binding protein C	MBL2_BOVIN	26	2.1	–	1.5	–	–
38	Fibrinogen alpha chain	FIBA_BOVIN	67	0.7	–	1.5	–	–
39	Protein AMBP	AMBP_BOVIN	39	1.4	8.6	2.0	–	–
40	Alpha-1-acid glycoprotein	A1AG_BOVIN	23	–	–	1.0	–	–
41	Transthyretin	TTHY_BOVIN	16	–	–	1.5	–	–
42	Clusterin	CLUS_BOVIN	51	–	–	0.5	–	–
43	Moesin	MOES_BOVIN	68	–	–	1.5	–	–
44	Complement factor B	CFAB_BOVIN	85	1.4	–	0.5	–	–
45	Adenylyl cyclase-associated protein 1	CAP1_BOVIN	51	–	–	2.0	–	–
46	Proactivator polypeptide	SAP_BOVIN	58	–	–	0.5	–	–
47	Factor XIIa inhibitor	F12AI_BOVIN	52	1.4	–	–	–	–
48	Tetranectin	TETN_BOVIN	22	–	–	1.5	–	–

Bions were prepared by adding CaCl_2_, MnCl_2_, SrCl_2_, or BaCl_2_ at a final concentration of 10 mM, or MgCl_2_ at 20 mM in DMEM containing 5% FBS. Na_2_HPO_4_ at 10 mM was then added (in a final volume of 1 ml). Following incubation in cell culture conditions overnight, bions were washed and then treated with 0.1 mM EDTA to help release the bound proteins. Proteomic analysis was performed using LC-MS/MS as described in *Materials and Methods*. Spectral counting values were normalized by multiplying each number of spectra by the average of total spectra count for the five samples shown, and then dividing by the sum of spectra for the corresponding sample. MW, molecular weight in kDa.

A total of 48 serum proteins were identified within the five bion species examined (Table 1). Every bion sample was shown to contain fetuin-A and albumin as the two main proteins identified. These two proteins represent calcification inhibitors that prevent ectopic calcification in vivo, and they have been consistently associated with mineral NPs in previous studies [1]–[5], [69], [86]. Accordingly, fetuin-A contains numerous calcium-binding [87] and HAP-binding sites [88], which are thought to be associated with its calcification inhibition activity [88], [89]. Albumin also contains calcium-binding sites [90], which may also explain its consistent interaction with mineral particles and its ability to inhibit calcification [91], [92]. In addition, the high abundance of fetuin-A and albumin in bions may reflect the high concentration of these proteins in serum: fetuin-A is present at 10–21 mg/ml in FBS [93] and at 0.7–0.8 mg/ml in HS [94], whereas albumin is found at 23 mg/ml [95] in FBS and at 35–45 mg/ml in HS [96]. Notably, bions derived from calcium, strontium, and barium appeared to bind more avidly to fetuin-A compared to the other bion samples, whereas bions obtained from magnesium and manganese seemed to bind to albumin in greater amounts than the other bions.

In addition to fetuin-A and albumin, other proteins were identified in all five bion samples studied here, including apolipoprotein A-I, prothrombin, complement C3, alpha-1-antiproteinase, vitamin K-dependent protein S, vitamin K-dependent protein C, adiponectin, and alpha-2-antiplasmin (Table 1). On the other hand, other proteins were found only in specific bion species, an observation which further supports the possibility that some proteins may bind specifically to a particular mineral phase. These results indicate that a large number of proteins covering a wide spectrum of biological functions can all associate with bions, with some binding specifically to the mineral phases while others do so only circumstantially due to abundance and availability. The bion-bound proteins identified here are thus similar to the ones found earlier for carbonate apatite NPs [7] and suggest that they may mirror the protein composition of the biological fluid in which the bions are assembled.

### Protein-Mineral Interactions: Albumin and Fetuin-A as Examples of both Inhibitors and Seeders of Bion Formation

We observed earlier [2], [4] that both albumin and fetuin-A could reproduce the dual inhibition-seeding mechanism of Ca-bion formation described for the whole serum. To evaluate whether albumin and fetuin-A inhibit bion formation produced by cations other than calcium, we selected one cation, barium, for analysis. For comparison, we also prepared Ca-bions under the same conditions. We induced bion formation by first adding 3 mM of either calcium or barium in DMEM containing albumin, fetuin-A, or both, followed by addition of 3 mM of phosphate. Bion formation was evaluated by monitoring turbidity as established previously for calcium-containing mineralo-organic NPs (Ca-bions) [2].

**Figure 9:**
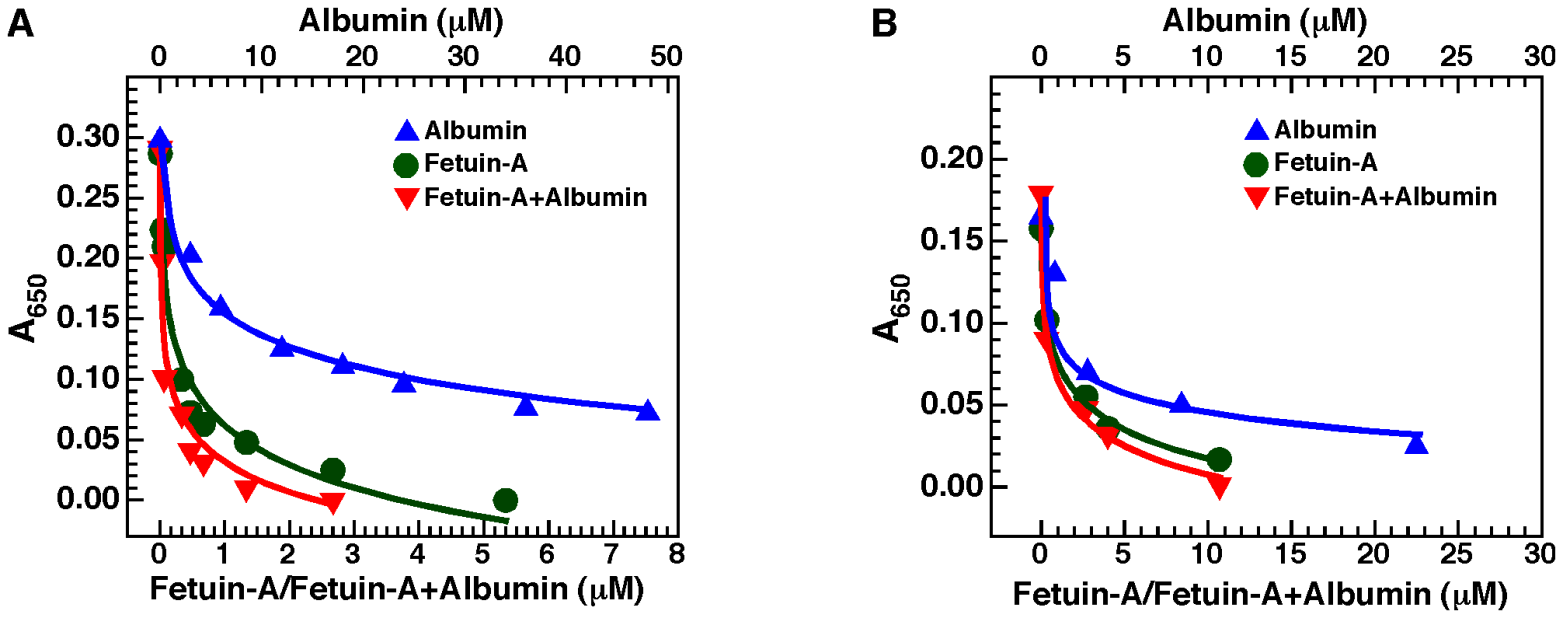
Albumin and fetuin-A inhibit bion formation. Bions were prepared by adding 3_2_ (A) or BaCl_2_ (B) in DMEM containing albumin and/or fetuin-A, followed by addition of 3 mM Na_2_HPO_4_ to induce bion formation. When used alone, albumin was added at the concentrations shown on top whereas fetuin-A was used at the concentrations shown at the bottom. When albumin and fetuin-A were added together, both proteins were used at the concentrations written at the bottom. Bion formation was evaluated by monitoring absorbance at a wavelength of 650 nm (A_650_). Compared to albumin, fetuin-A showed a more potent inhibitory effect on the formation of both types of bions. A slight additive inhibitory effect was noted when both albumin and fetuin-A were added together.

In line with results shown earlier [2], both albumin and fetuin-A inhibited Ca-bion formation when added alone in the precipitation mixture (Fig. 9A). Compared to albumin, fetuin-A was more efficient at inhibiting the formation of Ca-bions (Fig. 9A). When both albumin and fetuin-A were added together, a minor additive inhibitory effect was noted compared with the use of fetuin-A alone (Fig. 9A). Notably, similar results were obtained for bions prepared with barium instead of calcium (Fig. 9B). That is, both albumin and fetuin-A were shown to inhibit Ba-bion formation when added alone (Fig. 9B). In addition, fetuin-A was more efficient than albumin at inhibiting Ba-bion formation, and the use of both proteins together slightly enhanced the inhibitory effect of the proteins compared to their use alone (Fig. 9B). Overall, albumin and fetuin-A were shown to inhibit the formation of both Ca- and Ba-bions.

When the same precipitation mixtures were incubated for a few days under sterile cell culture conditions, we noticed that the level of Ba-bions increased with time (data not shown), a phenomenon also noted earlier for Ca-bions [2]. Mineral precipitation of Ba-bions occurred earlier and reached higher levels for albumin whereas it was delayed and was less abundant for the mixtures containing fetuin-A (data not shown), a result consistent with the higher mineralization-inhibitory potential of fetuin-A as compared to albumin (Fig. 9B). We presume that the initial inhibition produced by the two serum proteins is overcome with time, followed by the formation of particles containing the serum proteins embedded in the mineral phase. These experiments suggest further that, like the whole serum, albumin and fetuin-A may act as both inhibitors and seeders of bions.

### Increase in Size and Number, and Sub-Culture of Bions

One salient feature of the mineralo-organic NPs and complexes (i.e., Ca-bions) described earlier is their ability to mimick the increase in size and number, and the sub-culture of common microorganisms [1], [2], [5], [8]. In our hands [1]–[10], while Ca-bions represent lifeless biomimetic entities, they continue nonetheless to nucleate and grow in size presumably due to interactions between precipitating minerals and organic compounds. They ultimately give rise to new particles through secondary nucleation of mineral seeds that then interact further with organic moieties, a mechanism not dissimilar to the much better-known biomineralization process [97]. These biomimetic properties of Ca-bions explain a vast body of artifacts, controversies, and erroneous interpretations surrounding NB that have viewed the same cell-like properties of NPs (i.e., increase in size and number, and sub-culture) as *sine qua non* conditions indicative of life [1], [2], [8].

**Figure 10:**
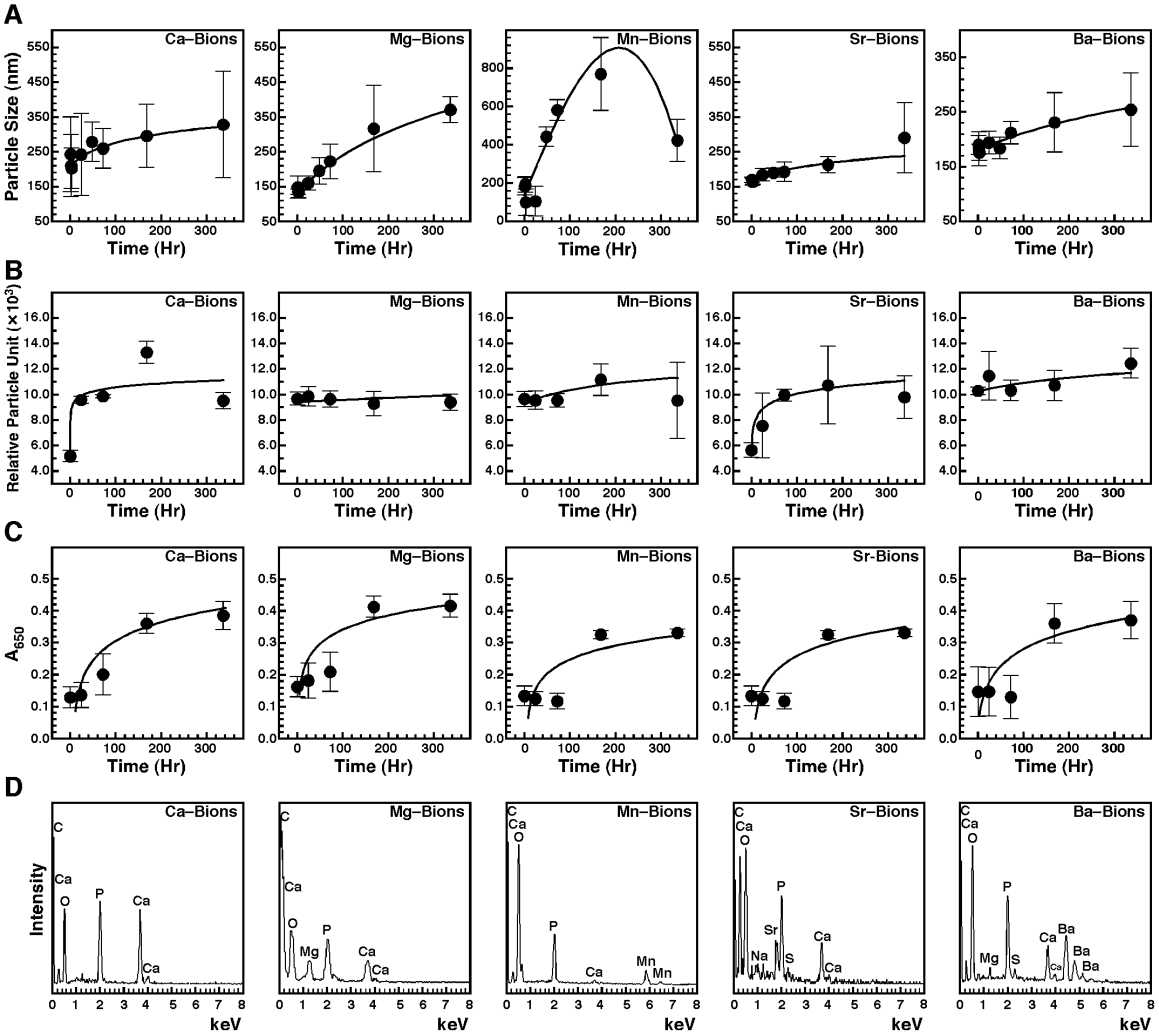
Increase in size and number, and sub-culture of bions. (A) Bions increase in size during incubation. Bions were prepared by adding 1 mM CaCl_2_, 10 mM MgCl_2_, 1 mM MnCl_2_, 5 mM SrCl_2_ or 3 mM BaCl_2_ in DMEM containing 5% FBS, followed by addition of Na_2_HPO_4_ at the same concentration to induce precipitation. Particle size was determined by DLS during a 2-week incubation period. (B) Bions increase in number in culture. Bions were prepared and incubated as described in (A) and particle number was determined by DLS. (C) Bions are sub-culturable in fresh medium. Bions were subcultured by diluting a solution containing abundant, pre-formed bions (A_650_ of 0.7) in fresh DMEM containing 10% FBS at a ratio of 1∶10 using a final volume of 15 ml. Bions sub-cultures were incubated in cell culture conditions for 2 weeks and the amount of bions was monitored by light absorbance. (D) EDX analysis of bions after sub-culture and incubation for 2 weeks. Bions were retrieved by centrifugation, followed by washing steps and preparation for EDX analysis. See the text for more details.

In order to verify whether bions obtained from cations other than calcium display similar biomimetic properties, we used dynamic light scattering (DLS) to monitor the size of nascent bions incubated in cell culture conditions (Fig. 10). In line with our previous results [10], mineralo-organic NPs (Ca-bions) prepared by the addition of 1 mM calcium and phosphate ions in DMEM containing 5% FBS can be shown to increase in size in a time-dependent manner during incubation (Fig. 10A). Likewise, bions prepared from other cations were also seen to increase in size during the same observation period (Fig. 10A; note however that Mn, Ba, Sr and Mg were added at the final concentrations of 1, 3, 5 and 10 mM, respectively, in order to obtain comparable levels of mineral particles for each ion). Notably and unlike the bions prepared using Mg, Sr, and Ba, which showed somewhat similar growth curves, the bions prepared using Mn produced a sharp initial increase in size up to 175 hrs, followed by a decrease in size thereafter (Fig. 10A). The diameter of the bions for all samples appeared to stabilize around 250–550 nm after the 2-week incubation time used for our studies (Fig. 10A). As seen from these results, bions formed from each one of the cations studied here increased in size as a function of time spent in culture.

We also examined the possibility that bions may increase in number during incubation in body fluids. Our previous studies [2]–[4], [6], [8], [10] had shown that mineralo-organic NPs (i.e., Ca-bions) can increase in number in supersaturated ionic solutions, a phenomenon that was attributed to the secondary nucleation of minerals known to occur during biomineralization [97]. Similarly, all bions tested here, except for those prepared with Mg, gradually increased in number in culture (Fig. 10B; note that bions were prepared as in Fig. 10A using different ion concentrations for each cation). The absence of increase for the number of Mg-bions—even though Mg was added at a concentration of 10 mM—was consistent with the predominantly inhibitory properties of magnesium on HAP formation as noticed earlier (Fig. 7A; see also ref. [2]). The differences in the curves representing the number of particles obtained for the various bions were attributed not only to different concentrations of cations used, but also the possibility that the increase in the number of bions may depend on the properties of the specific cation involved. Moreover, EDX and FTIR analyses of these bion samples after 2 weeks of incubation indicated that the particles still harbored carbonate HAP and contained the cation initially added in the mixture to induce bion formation (data not shown).

We also showed earlier that mineralo-organic NPs formed predominantly from calcium and phosphate precipitation (Ca-bions) can be sub-cultured in a manner similar to living microorganisms [2], [9], a phenomenon that was attributed to the ability of minerals to nucleate additional mineral particles by secondary nucleation [97]. Notably, bions prepared from various charged elements in DMEM containing 10% FBS were also sub-culturable from a 1:10 dilution of an abundant culture in a fresh culture medium with or without serum (Fig. 10C). Figure 10C shows a typical bion sub-culture experiment in which particle formation was monitored for 2 weeks using spectrophotometry. This sub-culture could be repeated several times for the various bions tested (data not shown). Still, the amount of precipitate gradually decreased with each sub-culture and by increasing the concentration of serum used, similar to previous results obtained with Ca-bions [2]. EDX and FTIR analyses confirmed that the sub-cultured bions contained calcium and phosphate ions as well as the cation initially added in the solution to induce bion formation (Fig. 10D). With longer incubation and through repeated sub-culture in DMEM containing serum, the cation initially added to induce bion formation was gradually replaced by calcium, indicating that the composition of the particles may be modulated by passages into fresh liquid medium.

Based on these observations, we conclude that bions prepared from various charged cations possess biomimetic properties similar to the Ca-enriched mineralo-organic NPs characterized earlier [2], [5], [9].

### Conclusion and Future Perspectives

The results presented in this study show that various elements and cations form biomimetic mineralo-organic NPs following precipitation with phosphate in biological fluids. We believe that these findings may have important implications for physiology and disease.

In the context of human physiology, the fact that bions spontaneously precipitate from elements and ions that are ubiquitous in biological fluids (e.g., Ca^2+^, PO_4_^3-^, Na^+^, Mg^2+^, Mn^2+^) suggests that the mineralo-organic particles may also form in the human body. Mineral NPs similar to Ca-bions have been shown to form in the growing bones and teeth of vertebrates, where they may represent precursors of mineralized organs [70], [98], [99]. In a previous study [3], we detected small amounts of mineral-protein NPs similar to Ca-bions following ultra-centrifugation of healthy human serum. Likewise, round and multilamellar mineral NPs and granules similar to bions have been repeatedly detected in the kidneys of healthy human individuals (reviewed by Ryall in ref. [11]). Human biological fluids are several orders of magnitude above supersaturation in terms of calcium and phosphate concentrations, indicating that mineral precipitation must continually be repressed in a systemic manner to avoid ectopic calcification in vivo [86], [100], [101]. These observations suggest that mineral particles similar to bions may form not only during normal mineralization processes in vivo but also at low levels in soft tissues as part of normal mineral and ionic homeostasis.

While bions may form in body fluids, several mechanisms exist in the body to protect these fluids and soft tissues against mineral precipitation and the accumulation of mineral NPs. Perhaps the most important mechanism is the regulation of mineral ions by the kidneys, lungs, and other organs, which help maintain ion concentrations within physiological ranges and thus prevents mineral precipitation in soft tissues. In addition, we have observed that serum proteins like albumin and fetuin-A inhibit the formation of bions in culture (Fig. 9; see also ref. [2]). These proteins, along with others (e.g., matrix Gla protein, osteopontin, osteoprotegerin), act as calcification inhibitors that prevent the formation and aggregation of mineral NPs and larger precipitates in body fluids [86], [100], [102], [103]. Jahnen-Dechent and colleagues have proposed that fetuin-A and other serum proteins may act as mineral chaperones that stabilize the formation of mineral NPs, which this group called calciprotein particles, thereby allowing the safe transport of mineral particles in a manner similar to lipid transport within lipoprotein particles [69], [86], [88], [101], [104]–[107].

In addition, a recent study [10] indicates that Ca-bions of various sizes can be phagocytized by macrophages in vitro, suggesting that these particles may be cleared by the reticuloendothelial system in the body. The work of Herrmann et al. [108] has recently confirmed this possibility by showing that fetuin-A-containing calciprotein particles injected intravenously in mice are rapidly cleared by macrophages located in the liver and spleen, a process which relied on the scavenger receptor-A present on macrophages. In health, these protective mechanisms may thus act together to prevent the formation of mineral NPs and remove any excess of such entities from body fluids.

As noted before, our initial interest in bions was triggered by past and still lingering controversy surrounding nanobacteria. Nanobacteria or NB have also been renamed calcifying nanoparticles (CNPs), a term which has removed the presumption that NB need to be alive [22], [30]–[34], [36], [109]–[114]. Nonetheless, CNPs have been shown to harbor nucleic acids [35], [110], [112], [114] as well as exotic bacterial proteins like translation elongation factor Tu and the molecular chaperone GroEL [35]. As shown by our studies, any milieu containing ions and organic moieties in excess will precipitate and the mineral precipitate will in turn undergo amorphous-to-crystalline phase transformation, a phenomenon which is independent of the presence of feeder serum or any other life-related processes [5], [6]. Our previous results [2], [5]–[7] further demonstrate that past proponents of NB/CNPs had in fact created *chimeric* NP entities containing both human protein antigens from diseased tissues as well as bovine proteins from the feeder serum, and after inoculating such NPs into a yet third unrelated animal species (e.g., rabbits), were making erroneous claims regarding *human* disease processes [30], [32], [115], [116]. Our results [1]–[6], [9] thus call into question the disease-related claims made earlier for NB/CNPs. Our studies also imply that the literature accumulated on this topic should be reinterpreted from a radically different point of view.

Based on the observations presented here and in our earlier studies, we believe that, contrary to previous claims [17], [18], [117]–[119], mineralo-organic NPs such as the bions presented here do not represent an infectious cause of disease but may instead be part of a physiological cycle that regulates the function, transport and disposal of mineral ions in the body [2], [5]. Accumulation of these particles may still occur and have pathological consequences in the body when calcium homeostasis is disturbed and when clearance mechanisms are overwhelmed. Accordingly, we have detected the presence of mineralo-protein NPs similar to Ca-bions in kidney and artery tissues obtained from human individuals suffering from various disease conditions (Wu et al., unpublished observations). Similarly, several studies have shown that mineral NPs similar to Ca-bions are found in ectopic calcifications in vertebrates [11], [68], [69], [120], [121]. These mineral NPs may be considered as precursors of calcium deposits that form in the body, including kidney stones and other calcification products found in the blood vessels of patients with atherosclerosis and kidney failure. Given that ectopic calcification is associated with increased mortality in patients with atherosclerosis and kidney failure [14], [122], determining the role that these particles play in disease processes has become a high priority from a medical point-of-view. This pathophysiological scenario however is radically different from the one based on the unsubstantiated claims advocated by the many NB/CNP studies published to date.

From another perspective, several ions that form bions in the present study, such as aluminum, cobalt, nickel, strontium and barium, are found as contaminants in the environment, and for this reason may interact with human biological fluids under various circumstances. Some of these elements, such as cobalt and nickel, have well-known toxicities when present at high concentrations in the human body [123], [124]. Recent studies suggest that mineral particles consisting of various minerals and heavy metals may form and accumulate in the tissues of healthy humans [125]–[127]. The possibility that these mineral NPs may disrupt biological processes and participate in degenerative conditions and disease is currently a topic of intense investigation. Based on these observations, Gatti and colleagues have proposed that the accumulation of mineral NPs in the human body may induce chronic inflammation and even cancer in genetically-susceptible individuals [127], [128]. These and other authors have used the term “nanopathology” to refer to the complications that could be caused by the accumulation of toxic mineral NPs in the human body [128]. Whether these mineral NPs play a role in human disease is a possibility that remains to be investigated.

We have observed recently that calcium phosphate NPs equivalent to Ca-based bions are phagocytized by macrophages irrespective of particle size, but that only large micro-particles or micrometer-sized NP aggregates may induce pro-inflammatory responses in these cells [10]. These unexpected results have recently been given support by Smith et al. [129], who showed that calcium phosphate NPs containing fetuin-A induce lower secretion of pro-inflammatory cytokines and have lower pro-apoptotic effects in macrophages compared to naked apatite crystals. Nonetheless, these results contrast with previous studies, which have reported that the pro-inflammatory effects of synthetic nanomaterials may be inversely correlated with particle size [130], [131]. It thus appears that synthetic and biological NPs may produce different effects on human cells and tissues.

The formation of bions may also have implications for understanding the effects of novel synthetic nanomaterials on cells and in the human body within the context of nanotoxicology. Previous studies have shown that man-made nanomaterials that enter the body rapidly become coated by a layer of proteins termed the protein corona [61], [63], [66], [67]. The nature of the protein corona is thought to influence the effects of nanomaterials on cells and organs as well as their distribution in vivo. We have found that several serum proteins interact with bions (Fig. 8), and that bions containing a particular element and mineral may bind to specific proteins from the serum (Table 1). These proteins may help us identify in more detail the effects of bions on cells and organs of the human body. For instance, given that bions harbor opsonin proteins like fetuin-A, complement proteins, and antibodies (which all enhance phagocytosis by immune cells [132]–[134]) as well as anti-opsonins like albumin (which represses phagocytosis [132]), the clearance of bions in vivo may be determined in part by the nature and amount of such proteins on the particles’ surface.

In a broader sense, we have found that bions prepared from various elements possess biomimetic properties (Figs. 1, 2, 3, 4, and 10) similar to the calcium phosphate particles that were characterized earlier [2], [8], [10]. Our results indicate that various minerals may form round, bacteria-like NPs when they precipitate in aqueous environment in the presence of sufficient amounts of calcification inhibitors, suggesting that these mineral NPs may be found in a wide range of environments and conditions. This observation suggests that great caution should be used while searching for living organisms in samples of unknown nature or origin. We have shown previously [9] that the mineral nano-entities seen in the Martian meteorite ALH84001 resemble the biomimetic mineral NPs that form in body fluids. With the continued search for extraterrestrial life and the recent landing of NASA’s Curiosity rover on Mars [135], our repeated observations that morphology is a poor criterion as proof of life should be considered highly relevant in future studies (Figs. 1, 2, 3, 4; see also ref. [9]). A similar note of caution was also expressed previously by other authors, most prominently by Garcia-Ruiz and colleagues [136]–[139], who have elegantly demonstrated that simple minerals like barium carbonate and silica can form biomimetic NPs reminiscent of living microorganisms.

With regards to their biological cycle, bions appear to share several characteristics with prions which are widely viewed as causing transmissible spongiform encephalopathy (TSE), a class of fatal neurodegenerative diseases affecting humans and mammals [140], [141]. Misfolded prions (PrP^Sc^) represent infectious proteinaceous agents that can replicate when seeded into a solution containing sufficient amounts of the normal cellular prion protein (PrP^C^). Similarly, we observed that bions can increase in number when an aliquot of particles is seeded into a solution containing sufficient precipitating ions (Figs. 7 and 10). Another point of similarity with prions may be the endogenous nature of both prions and bions. In the case of prions, the normal cellular form of the prion protein is thought to play a role during normal neurophysiology, but it may become infectious following a conformational change believed to be induced by mutation or contact with exogenous PrP^Sc^ [140], [141]. Similarly, bions may form in vivo from dissolved ions, proteins and organic molecules due to various factors as described above. In terms of potential infectivity, injection of misfolded prions (PrP^Sc^) into healthy animals harboring the normal PrP^C^ protein or contact between normal tissues and exogenous PrP^Sc^ may result in development of TSE [140], [141]. For bions, however, we believe that these particles are part of a normal biological cycle of storage, retrieval, and disposal that at times gets overwhelmed when there is excess of precipitating ions in the body fluid or cell milieu which in turn results in mineralization. In this sense, unlike prions, bions can not be deemed as infectious agents *per se* but they may certainly result in stone-related pathologies when they accumulate in the body.

We observed that the bions described here also share some characteristics with the formation of polymer gels obtained following incubation of ocean water at 20°C for several days [142]. Filtered ocean water yields a network of organic matter interspersed with colloid particles and calcium carbonate crystals that gradually increase in size during incubation. In that study, the formation of carbonate crystals was attributed to a Donnan equilibrium effect since the concentration of carbonate and calcium ions within the organic matrix may have increased compared to the outside water environment due to the polyanionic and semi-porous nature of the matrix. Our results suggest that a similar phenomenon involving interactions between dissolved ions and organic molecules may be at play during the formation of bions in biological fluids.

Overall, our observations indicate that the formation of bions is associated with the formation of an amorphous mineral phase, which is partially stabilized by calcification inhibitors—mainly organic molecules—present in body fluids. These mineralo-organic NPs possess unique morphologies and properties that mimick living organisms, and should have important implications in the fields of biomineralization, medicine, nanotoxicology, nanotechnology, and human health.

## Materials and Methods

### Formation of Bions in Body Fluids

HS, saliva and urine were collected from fasting healthy volunteers as described previously [2]. Ascites, cerebrospinal fluid, pleural effusion, and synovial fluid were obtained from patients with various clinical conditions as before [2]. Broncho-alveolar lavage was collected using standard procedures from patients with suspected lung conditions (Chang Gung Memorial Hospital, Linkou, Taiwan). The use of human samples in this study was approved by the Institutional Review Board of Chang Gung Memorial Hospital. Written informed consents were obtained from the volunteers who provided body fluid samples. FBS was obtained from PAA Laboratories (Pashing, Austria). All body fluids were filtered successively through both 0.2 and 0.1-µm pore membranes (Millipore, Billerica, MA, USA) prior to use. Stock solutions of NaCl, MgCl_2_, AlCl_3_, CaCl_2_, MnCl_2_, FeCl_2_, CoCl_2_, NiCl_2_, CuCl_2_, ZnCl_2_, SrCl_2_ and BaCl_2_ were prepared at 0.25 M with double-distilled water. The pH of the ion solutions was adjusted to 7.4 with 10 mM NaOH. Solutions of Na_2_HPO_4_/NaH_2_PO_4_ (0.25 M, pH 7.4) were prepared as before [2]. Unless specified otherwise, reagents and chemicals were purchased from Sigma (St. Louis, MO, USA). All element and ion solutions were filtered through a 0.1-µm pore membrane prior to use.

Bions were prepared by successively adding 1-10 mM each of the indicated element and Na_2_HPO_4_ to DMEM (Gibco, Carlsbad, CA, USA) containing a body fluid at 0.1–10%. In some experiments, 1 mM Na_2_CO_3_ was also added into DMEM prior to addition of Na_2_HPO_4_. Following incubation in cell culture conditions (37°C, 5% CO_2_, humidified air), bions were pelleted by centrifugation in a micro-centrifuge at 16,000×*g* for 10 min at room temperature, and washed twice with HEPES buffer (20 mM, pH 7.4) using the same centrifugation steps. Pelleted bions were finally used for analysis as described below. Prior to the addition of precipitating ions, the DMEM used in our experiments contained the following ions: Ca^2+^, 1.8 mM; Cl^−^, 121.5 mM; CO_3_^2−^, 44.1 mM; Fe^3+^, 0.0002 mM; K^+^, 5.3 mM; Mg^2+^, 0.8 mM; Na^+^, 157.9 mM; NO_3_^−^, 0.0002 mM; PO_4_^3−^, 0.9 mM; SO_4_^2−^, 0.8 mM. In experiments in which prolonged incubation in cell culture conditions was required, 0.02% sodium azide was added to prevent microbial contamination. All experiments were performed in triplicate.

In some experiments, the charged elements and phosphate were added in double-distilled water containing or not a body fluid at 0.1–10% to verify the role of body fluids and cell culture medium on bion formation. Bions were subcultured by diluting one volume of an abundant culture (corresponding to 15 ml of a solution with an optical density of 0.7 at 650 nm) in nine volumes of fresh cell culture medium containing or not 10% serum (final volume of 15 ml), prior to incubation in cell culture conditions for one month.

### Electron Microscopy

For electron microscopy, bions were prepared by diluting FBS, HS, saliva or urine at 10% into DMEM, followed by addition of 1 mM of the indicated charged element and phosphate, followed by incubation overnight (or as indicated) in cell culture conditions. For SEM, pelleted bions were re-suspended in double-distilled water and deposited on formvar carbon-coated grids. Excess liquid was removed with an absorbent paper and the grids were dried overnight under a laminar flow hood. The grids were coated with gold for 90 sec. Specimens were observed with a field emission SEM S-5000 scanning electron microscope (Hitachi Science Systems, Tokyo, Japan).

For thin-section TEM, pelleted bions were dehydrated with two washes of 100% ethanol. The samples were then embedded in Epon 812 resin (Electron Microscopy Sciences, Fort Washington, PA, USA) and incubated at 72°C for two days to allow resin polymerization. Thin sections were cut using a Leica Ultracut UCT microtome (Leica Microsystems, Wetzlar, Germany) and then transferred on formvar carbon grids. Thin section specimens were observed without staining under a JEOL JEM-1230 TEM (JEOL, Tokyo, Japan) operated at 120 keV.

### Spectroscopy Analyses

Washed bions were subjected to EDX, FTIR and powder XRD analyses as described previously [2], [3]. For EDX, aliquots of washed bions resuspended in water were deposited on formvar carbon-coated grids and dried overnight. EDX spectroscopy was performed without gold coating using a SEM S-3000N microscope (Hitachi Science Systems) equipped with an EMAX Energy EX-400 EDX device (Horiba, Tokyo, Japan).

For FTIR, dried bion powder was mixed 1:100 (w/w) with KBr and compressed into a 1.3-cm diameter pellet using a hand press. FTIR spectra were obtained using a Nicolet 5700 FTIR spectrophotometer (Thermo Fisher Scientific, Waltham, MA, USA). The following commercial reagents were used: calcium carbonate (Mallinckrodt Baker, Phillipsburg, NJ, USA), calcium phosphate tribasic (Kanto Chemical Corporation, Tokyo, Japan) and HAP (buffered aqueous suspension, 25% solid, Sigma). XRD analysis was performed as before [2], [4].

Powder XRD analysis was performed as before [4]. Briefly, washed bion samples were deposited on glass slides and dried overnight. XRD spectra were obtained with a D5005 X-ray diffractometer (Bruker AXS, Madison, Wi, USA). Spectra were compared with the database of the Joint Committee on Powder Diffraction and Standards (JCCDS).

### Dual Inhibition-Seeding Assay

Mineral particles were prepared in 24-well plates (Costar 3524, Corning Incorporated, Corning, NY, USA) as described before [3], [4]. Stock solutions of CaCl_2_, MgCl_2_, MnCl_2_, SrCl_2_, or BaCl_2_, (0.25 M, pH 7.4) were diluted at a final concentration of 10 mM in DMEM containing 0.1–10% FBS. After gentle shaking, Na_2_HPO_4_ was added into each well to a final concentration of 10 mM. Sodium azide was added to 0.02%. The final volume of reaction mixture was 1 ml in each well. The plates were incubated at 37°C for at least one month in cell culture conditions. Images of the 24-well plates were obtained using a scanner in reflective light mode (Scan Maker 8700, MicroTek, Hsinchu, Taiwan) as described earlier [2]. Photography and A_650_ turbidity readings referred to as “Day 1” were taken within one hour of reagent mixing. The data were presented as means ± standard deviation of five independent experiments. Mean comparisons were analyzed using Student’s *t* test. *P* values below 0.05 were considered statistically significant.

### SDS-PAGE and Proteomic Analysis

Bions were prepared by adding 10 mM CaCl_2_, MnCl_2_, SrCl_2_, or BaCl_2_, or 20 mM of MgCl_2_ in 1 ml of DMEM containing 5% FBS. Then, 10 mM of Na_2_HPO_4_ was added in a final volume of 1 ml. After incubation in cell culture conditions for two days, the particles were collected by centrifugation at 16,000×*g* for 10 min and washed twice with HEPES buffer using the same centrifugation steps, prior to resuspension in 200 μl of HEPES buffer. Fifty μl of EDTA (0.5 mM, pH 8) was added to partially dissolve the particles and release bion-associated proteins. The supernatants were collected after centrifugation and 15 μl of samples was loaded and analyzed by SDS-PAGE under denaturing and reducing conditions [7].

The proteomic analysis was performed as before [7]. Twenty μl of the samples SDS-PAGE described above was used for in-solution trypsin digestion and LC-MS/MS analysis. Briefly, MS/MS spectra were searched against the SwissProt database using the Mascot software (version 2.2, Matrix Science, Boston, MA, USA) with a fragment ion mass tolerance of 0.5 Da and a parent ion tolerance of 10 ppm. Mascot search results were integrated into the Scaffold software (Proteome Software, Portland, OR, USA) to obtain spectrum counts, sequence coverage, and the numbers of unique peptides. The criteria for protein identification included a minimum of two unique peptides identified with a Scaffold probability of 100%.

### Dual Inhibition-Seeding of Bions by Albumin and Fetuin-A

Stock solutions of human serum albumin or bovine serum fetuin-A (Sigma) were prepared in double-distilled water at a final concentration of 20 mg/ml. Albumin and fetuin-A were first added to water at the indicated concentrations prior to addition of either CaCl_2_ or BaCl_2_ at a final concentration of 3 mM each. Na_2_HPO_4_ was then added at 3 mM to induce bion formation. A final volume of 1 ml was used. Turbidity was determined at 650 nm with a Spectra Max M2 spectrophotometer (Molecular Devices, Sunnyvale, CA, USA) [2]. After spectrophotometric measurements, the solutions were incubated in cell culture conditions for various periods of time.

### DLS Analysis

Bion samples were prepared by adding CaCl_2_, MgCl_2_, MnCl_2_, SrCl_2_, or BaCl_2_, into DMEM containing 5% FBS and 0.02% sodium azide. To prepare abundant bions, the final concentration of ion solution used was 1 mM for Ca-bions, 5 mM for Sr-bions, 3 mM for Ba-bions, 1 mM for Mn-bions, and 10 mM for Mg-bions. Fifteen-ml centrifugation tubes were used (TPP Techno Plastic Products, Trasadingen, Switzerland). Sizing and counting of bions was performed as before [10] using a Coulter N4 Plus submicron particle analyzer (Beckman Coulter, Brea, CA, USA). Briefly, bion samples were incubated in cell culture conditions for various peri­ods of time and then 1 ml of bion solution was transferred to dis­posable plastic cuvettes (Kartell, Milan, Italy), prior to gentle mixing by inversion and reading. Detection was performed at room temperature at an incident angle of 90°. The relative particle unit corresponds to a relative value that correlates in a linear manner with the observed number of particles as observed under TEM.
