# Correction: Bions: A Family of Biomimetic Mineralo-Organic Complexes Derived from Biological Fluids

**DOI:** 10.1371/journal.pone.0091496

**Published:** 2014-03-21

**Authors:** The PLOS ONE Staff

There is an error in the Sr-Bions graph of Figure 10C. Please see the corrected Figure 10 here.

**Figure 10:**
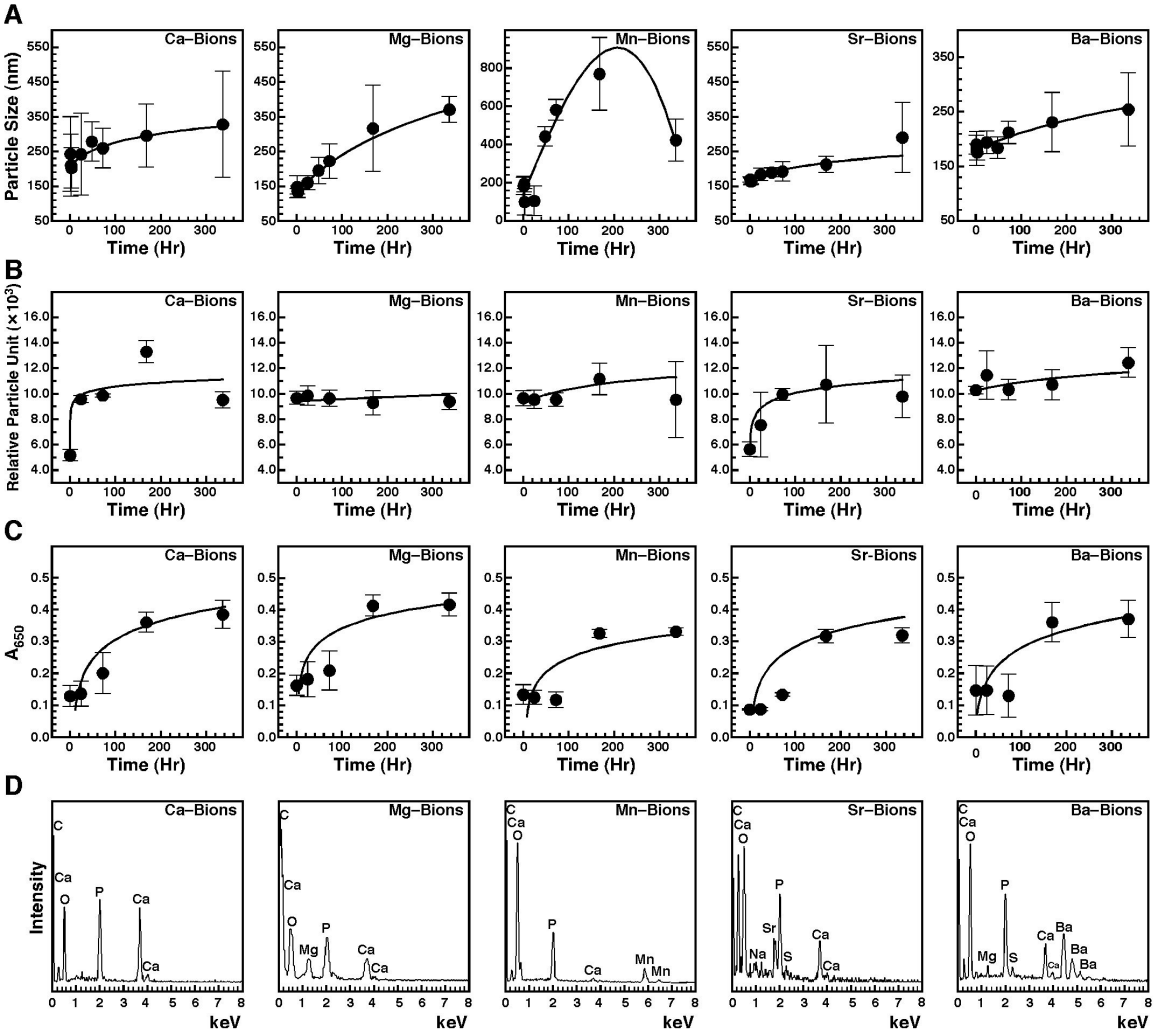
Increase in size and number, and sub-culture of bions. (A) Bions increase in size during incubation. Bions were prepared by adding 1 mM CaCl_2_, 10 mM MgCl_2_, 1 mM MnCl_2_, 5 mM SrCl_2_ or 3 mM BaCl_2_ in DMEM containing 5% FBS, followed by addition of Na_2_HPO_4_ at the same concentration to induce precipitation. Particle size was determined by DLS during a 2-week incubation period. (B) Bions increase in number in culture. Bions were prepared and incubated as described in (A) and particle number was determined by DLS. (C) Bions are sub-culturable in fresh medium. Bions were subcultured by diluting a solution containing abundant, pre-formed bions (A_650_ of 0.7) in fresh DMEM containing 10% FBS at a ratio of 1?10 using a final volume of 15 ml. Bions sub-cultures were incubated in cell culture conditions for 2 weeks and the amount of bions was monitored by light absorbance. (D) EDX analysis of bions after sub-culture and incubation for 2 weeks. Bions were retrieved by centrifugation, followed by washing steps and preparation for EDX analysis. See the text for more details.
